# Fracture of the Os Inominata

**Published:** 1883-04

**Authors:** Frank V. Walton


### XII.—FRACTURE OF THE OS INOMINATA.

BY FRANK V. WALTON, D.V.S.

This remarkable case was brought to the hospital in an ambulance, being absolutely unable to stand.

The history accompanying the case was that the day previous while on the road the animal caught one of his hind feet in a car track in some unexplainable manner, and in trying to extricate the foot tore off the shoe. The shoe was replaced, and he finished his day's work as usual. The next day he started out apparently as sound as ever, but about 11 o'clock, A. M., while pulling a load of sand, he suddenly staggered and fell.

When first seen an examination was made per rectum, and fracture of the pelvic bones diagnosticated. The fracture involved both isteum and pubis. In other words the line of fracture was across the anterior rami of the isteum into the obturator foramen, and from the obturator foramen through the transverse rami of the pubis into the pelvic cavity.

Prior to destruction the animal thrashed around considerably, and several of the prominent spines of the pelvic bones were found to be injured at the necropsy.

The treatment consisted in destruction of the animal.

[This certainly is a very interesting case, and raises the question as to the time the fracture was produced. Was it by the catching of the foot, or was there a diseased condition of the bone which rendered them unable to sustain the pressure of the heavy draught strain? We are inclined to the opinion that there must have been primarily a diseased condition of the bones, which rendered them more friable than normal, and that the unusual strain from catching the foot, followed by the heavy draught strain, finally resulted in the fracture. Had the symptoms developed immediately after the catching of the foot it might be looked upon as the sole cause.

Little else could be done in the way of treatment.

Had the animal been a valuable breeding mare the question of treatment might be worthy of consideration, but even then for only a moment. If the predisposing cause were a diseased condition of the bone the value for breeding purposes would be rapidly diminished. Again, if such a condition were treated, there would be great danger of a deformed pelvis and an obstruction to free delivery produced, so that in all cases death of the animal would be the only rational mode of dealing with such cases.—ED.]

